# *International Journal of Molecular Science* 2018 Best Paper Award

**DOI:** 10.3390/ijms19113694

**Published:** 2018-11-21

**Authors:** International Journal of Molecular Science Editorial Office

**Affiliations:** MDPI, St. Alban-Anlage 66, 4052 Basel, Switzerland; ijms@mdpi.com

The Editors of the *International Journal of Molecular Sciences* have established the Best Paper Award to recognize the most outstanding articles published in the areas of molecular biology, molecular physics, and chemistry that have been published in the *International Journal of Molecular Sciences*. The prizes have been awarded annually since 2012 [1,2,3,4,5,6].

We are pleased to announce the “*International Journal of Molecular Science* Best Paper Award” for 2018. Nominations, chosen from all papers published in 2017, were made by the Editorial Board. The awards are issued to reviews and research articles separately. Following a review process by the Editorial Board, three top-voted research articles and the three top-voted reviews, as follows in no particular order, have won the “*International Journal of Molecular Science* Best Paper Award” for 2018:

**Research Article Award:**

**Magnetic Beads-Based Sensor with Tailored Sensitivity for Rapid and Single-Step Amperometric Determination of miRNAs**

Eva Vargas, Rebeca M. Torrente-Rodríguez, Víctor Ruiz-Valdepeñas Montiel, Eloy Povedano, María Pedrero, Juan J. Montoya, Susana Campuzano and José M. Pingarrón

*Int. J. Mol. Sci.***2017**, *18*(11), 2151; doi:10.3390/ijms18112151

Available online: https://www.mdpi.com/1422-0067/18/11/2151

This paper describes the development of an amperometric biosensor for the sensitive determination of microRNAs using a single-step rapid protocol. This novel biosensing approach involves the use of direct hybridization of the target miRNA (miRNA-21) onto streptavidin-modified magnetic beads (MBs) where a specific biotinylated DNA probe is attached, and the labeling of the resulting heteroduplexes with a specific DNA–RNA antibody and the bacterial antibody-binding protein A (ProtA) conjugated with a homopolymer containing multiple horseradish peroxidase (HRP) molecules (ProtA-Poly-HRP40), as an enzymatic label for signal amplification. The magnitude of the cathodic current obtained at −0.20 V (versus the Ag pseudo-reference electrode) by performing amperometric detection, upon magnetic capture of the modified MBs onto the working electrode surface of disposable screen-printed carbon electrodes (SPCEs) in the presence of the H_2_O_2_–hydroquinone (HQ) system, demonstrated linear dependence with the concentration of the synthetic target miRNA over the 1.0 to 100 pM range. The developed biosensor provided a detection limit (LOD) of just 10 attomoles (in 25 μL sample) for the synthetic target miRNA, without any target miRNA amplification, and offered the possibility to tailor the sensitivity by enlarging the length of the DNA–miRNA heteroduplexes using additional probes and/or performing the labelling with ProtA conjugated with homopolymers prepared with different numbers of HRP molecules. Moreover, the practical usefulness of this biosensor was demonstrated by determining the endogenous levels of the mature target miRNA-21 in 250 ng of raw total RNA extracted from breast cancer cells and tumor tissues within 30 min, without requiring previous reverse transcription of RNA to cDNA, complex amplification protocols, or the use of an internal reference, and using portable and affordable instrumentation. The short assay time, simplicity, and feasibility to tailor the final sensitivity to be applied for the determination of any target RNA and to perform multiple analyses in a single experiment, position this versatile methodology as a promising tool for high-throughput and simple miRNAs/RNAs bioanalysis, applicable to a broad range of settings (Figure 1).

**Inhibition of Autophagy Promotes Salinomycin-Induced Apoptosis via Reactive Oxygen Species-Mediated PI3K/AKT/mTOR and ERK/p38 MAPK-Dependent Signaling in Human Prostate Cancer Cells**

Kwang-Youn Kim, Kwang-Il Park, Sang-Hun Kim, Sun-Nyoung Yu, Sul-Gi Park, Young Woo Kim, Young-Kyo Seo, Jin-Yeul Ma and Soon-Cheol Ahn

*Int. J. Mol. Sci.***2017**, *18*(5), 1088; doi:10.3390/ijms18051088

Available online: https://www.mdpi.com/1422-0067/18/5/1088

Recently, the interplay between autophagy and apoptosis has become an important factor in chemotherapy for cancer treatment. Inhibition of autophagy may be an effective strategy to improve the treatment of chemo-resistant cancer by consistent exposure to chemotherapeutic drugs. However, no reports have clearly elucidated the underlying mechanisms. Therefore, in this study, we assessed whether salinomycin, a promising anticancer drug, induces apoptosis and elucidated potential antitumor mechanisms in chemo-resistant prostate cancer cells. Cell viability assay, Western blot, annexin V/propidium iodide assay, acridine orange (AO) staining, caspase-3 activity assay, determination of reactive oxygen species (ROS) production, and mitochondrial membrane potential analysis were performed. Our data showed that salinomycin alters the sensitivity of prostate cancer cells to autophagy. Pretreatment with 3-methyladenine (3-MA), an autophagy inhibitor, enhanced salinomycin-induced apoptosis. Notably, salinomycin decreased phosphorylated AKT and phosphorylated mammalian target of rapamycin (mTOR) in prostate cancer cells. Pretreatment with LY294002, an autophagy and PI3K inhibitor, enhanced salinomycin-induced apoptosis by decreasing AKT and mTOR activities and suppressing autophagy. However, pretreatment with PD98059 and SB203580, extracellular signal-regulated kinases (ERK) and p38 inhibitors, respectively, suppressed salinomycin-induced autophagy by reversing the upregulation of ERK and p38. In addition, pretreatment with *N*-acetyl-l-cysteine (NAC), an antioxidant, inhibited salinomycin-induced autophagy by suppressing ROS production. Our results suggest that salinomycin induced apoptosis, which was related to ROS-mediated autophagy through the regulation of the PI3K/AKT/mTOR and ERK/p38 MAPK signaling pathways.

**Development of An Impedimetric Aptasensor for the Detection of *Staphylococcus aureus***

Peggy Reich, Regina Stoltenburg, Beate Strehlitz, Dieter Frense and Dieter Beckmann

*Int. J. Mol. Sci.***2017**, *18*(11), 2484; doi:10.3390/ijms18112484

Available online: https://www.mdpi.com/1422-0067/18/11/2484

Aptamers are promising and powerful analytical tools, especially as receptors for biosensor applications. In the awarded paper, we developed an aptamer-based biosensor detecting the human pathogen *Staphylococcus aureus* using electrochemical impedance spectroscopy (EIS). The detection of *S. aureus* is important in many fields, for example, to control food quality, to detect mastitis in dairy cows, and to prevent the spreading of nosocomial infections. In the described aptasensor, the bacteria of a given sample bind to protein A-specific aptamers immobilized on a gold electrode. Protein A is located on the bacterial cell surface and is known as one of the virulence factors of *S. aureus*. The thiol-modified protein A-binding aptamers were co-immobilized with 6-mercapto-1-hexanol onto gold electrodes by self-assembly. As determined with quartz crystal microbalance experiments, an average density of 1.01 ± 0.44 × 10^13^ aptamer molecules per cm^2^ was achieved. The detection of the captured bacteria was based on the decrease of the electron transfer between electrode and the mediator ferri-/ferrocyanide in the solution measured by EIS. For *S. aureus*, a dynamic measurement range of 10^0^ and 10^5^ colony-forming units per ml and a detection limit of 10 colony-forming units per ml was reached. Moreover, the aptasensor was demonstrated to distinguish non-target bacteria such as *Staphylococcus epidermidis* and *Escherichia coli*. EIS as a measurement method has the advantage that the bound bacteria are not harmed and can be further investigated by, for example, a polymerase chain reaction for antibiotic resistance detection. This work shows the high potential of impedimetric aptasensors in future biosensing (Figure 2).

**Review Paper Award:**

**Exosomes: From Garbage Bins to Promising Therapeutic Targets**

Mohammed H. Rashed, Emine Bayraktar, Gouda K. Helal, Mohamed F. Abd-Ellah, Paola Amero, Arturo Chavez-Reyes and Cristian Rodriguez-Aguayo

*Int. J. Mol. Sci.***2017**, *18*(3), 538; doi:10.3390/ijms18030538

Available online: https://www.mdpi.com/1422-0067/18/3/538

Our increasing understanding of why cells release exosomes and their role in intercellular communication has revealed the very complex and sophisticated contribution of exosomes to health and disease. The aim of this “Exosomes: From Garbage Bins to Promising Therapeutic Targets” review is to reveal the emerging roles of exosomes in normal and pathological conditions and describe the controversial biological role of exosomes, as it is now understood, in carcinogenesis. We summarize what is known about exosome biogenesis and composition, the diversity of exosome functions, the bioactive roles of exosomes in the maintenance of normal physiology, the pathological roles of exosomes in the spreading of disease, as well as the important role of exosomes in cancer and immune modulation and how exosomes can inhibit the functions of CD8+ cytotoxic T cells and natural killer (NK) cells and inhibit the differentiation of precursors to mature antigen-presenting cells. We also describe the effects of exosomes on the development and progression of cancers, highlighting their role in the suppression of immune surveillance. In this respect, it has been indicated that cancer stem cells (CSC) or tumor cells secrete exosomes that can both block tumor growth by promoting antitumor immune responses and induce tumor growth by attenuating antitumor immunity, which is the case for exosomal PDL-1, or promote angiogenesis and/or metastases to distant tissues or organs. We also discuss the potential clinical applications of exosomes, especially as biomarkers and novel therapeutic agents. We provide a compendium of studies reported in the literature, trying to uncover the physiological role of the entirely new mode of cell–cell communication mediated by exosomes that may provide us with the tools to further improve anticancer therapeutics and cancer diagnostics (Figure 3).

**Mesenchymal Stem/Stromal Cell-Derived Extracellular Vesicles and Their Potential as Novel Immunomodulatory Therapeutic Agents**

Verena Börger, Michel Bremer, Rita Ferrer-Tur, Lena Gockeln, Oumaima Stambouli, Amina Becic and Bernd Giebel

*Int. J. Mol. Sci.***2017**, *18*(7), 1450; doi:10.3390/ijms18071450

Available online: https://www.mdpi.com/1422-0067/18/7/1450

Extracellular Vesicles (EVs), such as exosomes and microvesicles, are shed by all cell types and found in all body fluids. EVs transmit specific information from their cells of origin to specific target cells and are key factors in a novel form of intercellular communication. Depending on their origin, EVs can modulate immune responses and act either as pro-inflammatory (e.g., mature DC-EVs) or as anti-inflammatory agents [e.g., mesenchymal stem cell EVs (MSC-EVs) and many tumor cell-derived EVs]. Setting up standards and improving the characterization of EVs are our main goal and are important for any clinical approach. As we have treated an acute steroid-refractory Graft-versus-Host-Disease patient, we are aware of the complexity of MSC-EVs. In this context, we reviewed recently published in vivo models, which were treated with MSC-EVs. These disease models included rodent kidney, heart, liver, and brain injury models. A variety of different protocols for the production, characterization and application of MSC-EVs were used in these studies and are summarized in our article. The comparison of these studies revealed a lack of standardization, which is a broadly discussed issue in the field. Within this review, we point out the challenges and discuss possible solutions to build up standards for future studies (Figure 4).

In case you have further questions, please do not hesitate to get in contact with us!

**Melatonin, a Full-Service Anti-Cancer Agent: Inhibition of Initiation, Progression, and Metastasis**

Russel J. Reiter, Sergio A. Rosales-Corral, Dun-Xian Tan, Dario Acuna-Castroviejo, Lilan Qin, Shun-Fa Yang and Kexin Xu

*Int. J. Mol. Sci.***2017**, *18*(4), 843; doi:10.3390/ijms18040843

Available online: https://www.mdpi.com/1422-0067/18/4/843

There is highly credible evidence that melatonin mitigates cancer at the initiation, progression, and metastasis phases. In many cases, the molecular mechanisms underpinning these inhibitory actions have been proposed. What is rather perplexing, however, is the large number of processes by which melatonin reportedly restrains cancer development and growth. These diverse actions suggest that what is being observed are merely epiphenomena of an underlying more fundamental action of melatonin that remains to be disclosed. Some of the arresting actions of melatonin on cancer are clearly membrane receptor-mediated, while others are membrane receptor-independent and involve direct intracellular actions of this ubiquitously distributed molecule. While the emphasis of melatonin–cancer research has been on the role of the indoleamine in restraining breast cancer, this is changing quickly, with many cancer types having been shown to be susceptible to inhibition by melatonin. There are several facets of this research which could have immediate applications at the clinical level. Many studies have shown that melatonin’s co-administration improves the sensitivity of cancers to inhibition by conventional drugs. Even more important are the findings that melatonin renders cancers that were previously totally resistant to treatment sensitive to these same therapies. Melatonin also inhibits molecular processes associated with metastasis by limiting the entrance of cancer cells into the vascular system and preventing them from establishing secondary growths at distant sites. This is of particular importance, since cancer metastasis often significantly contributes to the death of cancer patients. Another area that deserves additional consideration is related to the capacity of melatonin to reduce the toxic consequences of anti-cancer drugs while increasing their efficacy. Although this information has been available for more than a decade, it has not been adequately exploited at the clinical level. Even if the only beneficial actions of melatonin in cancer patients are its ability to attenuate acute and long-term drug toxicity, melatonin should be used to improve the physical wellbeing of the patients. The experimental findings, however, suggest that the advantages of using melatonin as a co-treatment with conventional cancer therapies would far exceed the improvements in the wellbeing of the patients. 

We believe that these six exceptional papers are valuable contributions to the *International Journal of Molecular Science* and the scientific research field. On behalf of the *International Journal of Molecular Science* Editorial Board, we would like to congratulate these teams for their excellent work. In recognition of their accomplishment, they will receive the privilege of publishing an additional research article or review paper free of charge in open access format in the *International Journal of Molecular Science*, after the usual peer-review procedure.

We would like to take this opportunity to thank all the nominated research groups of the above exceptional papers for their contributions to the *International Journal of Molecular Science* and thank the *International Journal of Molecular Science* Editorial Board for voting and helping with this “Best Paper Award”.

The Editorial Board and Editorial Staff at the *International Journal of Molecular Science* are committed to meeting the needs of the molecular research community by providing useful and timely reviews of all manuscripts submitted and an open access journal for results’ publication. Please consider submitting your work to the *International Journal of Molecular Science*. We look forward to announcing your paper as an *International Journal of Molecular Science* “Best Paper” in the future.

## Prize Awarding Committee

*International Journal of Molecular Science* Editorial Board.

## Figures and Tables

**Figure 1:**
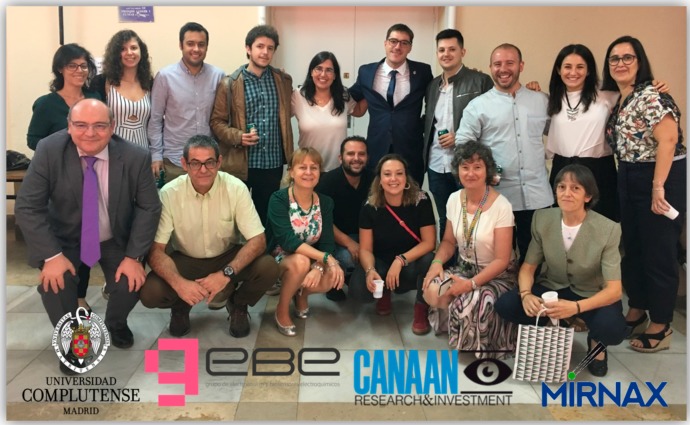
Electrochemistry and Electrochemical (Bio)sensors research Group, Analytical Chemistry Department, Faculty of Chemistry, University Complutense of Madrid (Spain) in collaboration with CANNAN RESEARCH & INVESTMENT S.L. and Mirnax Biosens S.L.

**Figure 2:**
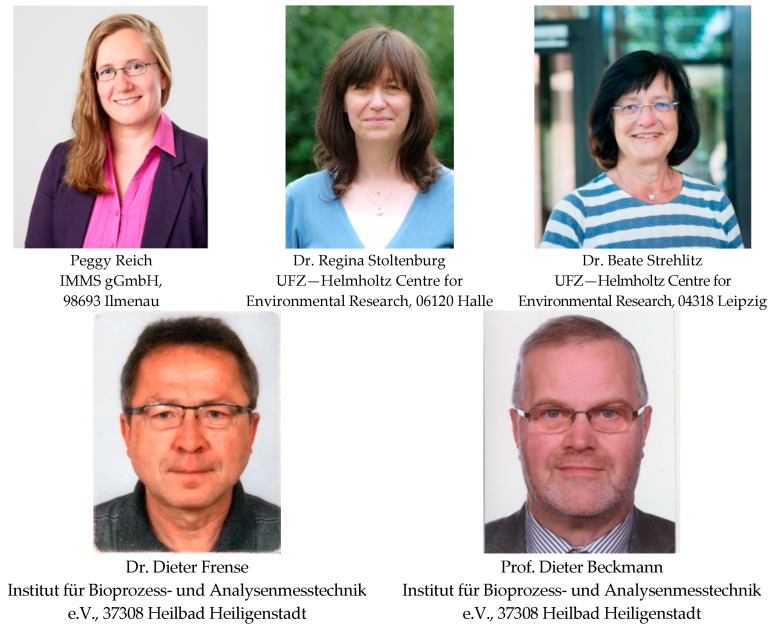
Dr. Reich’s research group.

**Figure 3:**
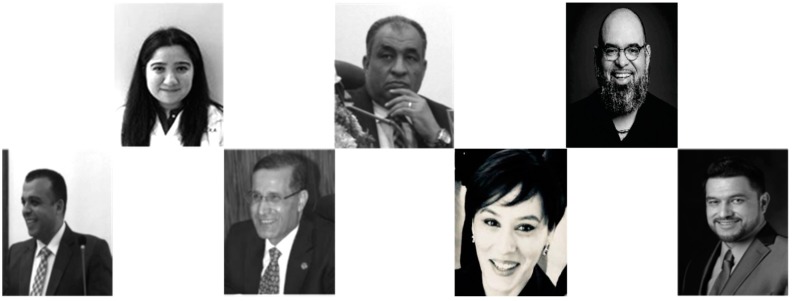
From left to right: Mohammed H. Rashed, Emine Bayraktar, Gouda K. Helal, Mohamed F. Abd-Ellah, Paola Amero, Arturo Chavez-Reyes, and Cristian Rodriguez-Aguayo.

**Figure 4:**
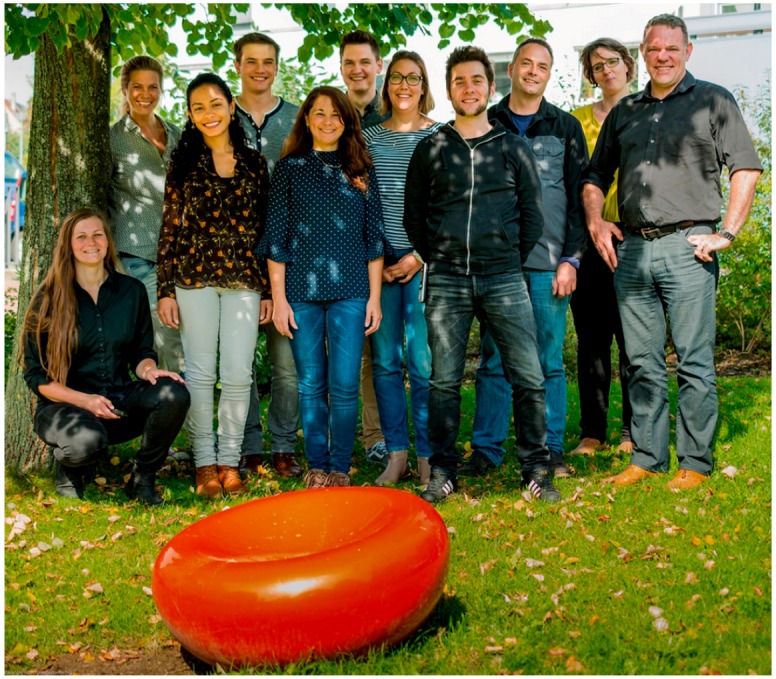
Dr. Börger’s research group.
